# Comparison of Serum Inhibin B and Follicle-Stimulating Hormone (FSH) Level between Normal and Infertile Men in Yaoundé

**DOI:** 10.1155/2020/4765809

**Published:** 2020-01-23

**Authors:** Tchoula Mamiafo Corinne, Pieme Constant Anatole, Ngogang Yonkeu Jeanne

**Affiliations:** ^1^Department of Biochemistry, Faculty of Sciences, University of Yaoundé I, PB 812 Yaoundé, Cameroon; ^2^Department of Biochemistry, Faculty of Medicine and Biomedical Sciences, University of Yaoundé I, PB 1364 Yaoundé, Cameroon

## Abstract

**Objective:**

Hormones play a vital role in initiating and maintaining male reproductive function. The present study explores the influence and predictive ability of two reproductive hormones on semen quality among men who were partners in an infertile couple.

**Design:**

During our cross sectional study, men were recruited from private and public hospital and laboratories for clinical evaluation of fertility status.

**Methods:**

Fresh semen samples were assessed for quality (concentration, motility and morphology) according to the 2010 World Health Organization manual and the serum levels of hormones, including follicle-stimulating hormone (FSH), Inhibin B was measured (ELISA).

**Results:**

We found a significant difference in the two groups regarding sperm concentration (*p* < 0.0001), total sperm count (*p* < 0.0001), total sperm count (*p* < 0.0001), total sperm count (*p* < 0.0001), total sperm count (*p* < 0.0001), total sperm count (

**Conclusion:**

Consistent with other studies, our results show strong association between semen quality and FSH and Inhibin B.

## 1. Introduction

Male infertility is found in 50% of infertile couples [1]. When reviewed, 55% of the reasons for infertility are found to be male-related and 35% to be female-related, while 10% constitutes infertility of unknown origin [2].

The etiology of declining male fertility can be related to falling androgen levels, decreased sexual activity, alterations in sperm motility and morphology, and deterioration in sperm quality and DNA integrity [3].

Previous studies have reported that circulating levels of specific reproductive hormones in men are associated with semen quality parameters [4]. In particular, Inhibin B and follicle-stimulating hormone (FSH) are thought to be markers of spermatogenesis and Sertoli cell function, and it has even been suggested that measuring the two hormones in serum could serve as a substitute for measuring semen quality or fecundability in epidemiologic studies [4].

Inhibin B is a heterodimeric glycoprotein composed of a common *α*-subunit and a specific *β*B-subunit [5]. In men, this protein is produced exclusively by the testis [5]. Inhibin B production is stimulated by the secretion of pituitary follicle-stimulating hormone (FSH). The resulting Inhibin B exerts a negative feedback on FSH secretion. Inhibin B also exerts a paracrine intratesticular effect [5]. The assessment of testicular function typically involves an initial endocrine assessment, in which serum FSH and total testosterone levels are routinely measured [6, 7]. When measured, Inhibin B levels correlate quite well with FSH concentrations in the vast majority of cases. However, the diagnostic accuracy of FSH is limited by the fact that some conditions do not lead to changes in FSH secretion [5]. FSH and Inhibin B are complementary tools in gonadal male conditions and andrological diagnostics [5, 8]. In particular, FSH and Inhibin B together are more sensitive than either alone in predicting the histological status of the testis and the presence of sperm in bioptic tissue [5].

This study is designed to study levels of follicle-stimulating hormone (FSH), and Inhibin B in blood serum, and their relation to seminal fluid functional parameters in a group of infertile patients.

## 2. Material and Methods

### 2.1. Subject Recruitment

The study population comprised healthy men referred for semen analysis to some private laboratories and health institutions. Our men subjects were members of infertile and fertile couples. For a given individual, the semen and blood samples were provided on the same day. All the participants gave their written informed consent.

### 2.2. Study Design

This cross-sectional study was carried out at six Public health institutions and Private laboratories of the Yaoundé Town. These centers are located in the political capital of Cameroon, and receive a large number of male patients living in Yaoundé and surrounding towns. A convenient sample of 240 infertile male patients, aged 18 years and above, were consecutively enrolled in the study during twelve months. But only 156 succeeded in providing both semen and blood samples. All the procedures used in this study were in accordance with the current revision of the Helsinki Declaration. A written and signed informed consent was provided by all the subjects. Consent forms and procedures, as well as survey protocol, were approved by the Cameroon National Ethics Committee (Reference number: 2015/04/572/CE/CNERSH/SP).

### 2.3. Interview Data

At enrollment standardized data collection forms were completed, including sociodemographic characteristics, the medical background, previous unions, chirurgical background, and sexuality.

### 2.4. Collection of Semen Samples

Semen was collected by masturbation into a sterile plastic specimen cup at the hospital or laboratory. Subjects were instructed to abstain from ejaculation for at least 72 hours prior to producing the semen sample. The actual abstinence period was recorded based on the information given by men at the time of the semen collection .The sample was liquefied for at least 30 minutes at 37°C, but no longer than 1 hour prior to performing a routine semen analysis, which included measurements of volume, pH, sperm concentration, sperm motility, progressive motility, and sperm morphology according to standard methodologies [9].

### 2.5. Evaluation of the Quality of Sperm

Before starting semen analysis, a period of time must be allowed for liquefaction. The normal liquefaction time is 30 minutes. If this is not done within this time, we will say that it is slow rather than normal. The semen volume, total sperm number, sperm concentration, motility, and vitality were interpreted according to the 2010 WHO guidelines.

Strict scoring criteria were used to classify men as having normal or subnormal morphology [9] as recommended by WHO guidelines. Kruger's classification identifies a single sperm anomaly. The anomaly is listed in order of importance as follows: acrosome, head, intermediate piece and flagella. As soon as an abnormality is identified, the spermatozoon is directly classified as “abnormal”.

### 2.6. Determination of Biochemical Parameters

Venous blood samples were taken from the cubital vein. The samples were left for 30 min at room temperature, then centrifuged at 3000 rpm for 15 minutes at room temperature. The sera were transported to small Eppendorf tubes and stored at −20°C. The serum Inhibin B was determined in duplicate using the Inhibin B Gen II ELISA technic of Beckman Coulter (250 S. Kraemer Blvd. Brea, CA 92821 U.S.A.) which is an enzymatically amplified three-step “sandwich” assay. In the assay, calibrators, controls and samples are incubated in microtitration wells which have been coated with anti-activin B antibody [10]. Serum FSH levels were measured with a Microplate Immunoenzymometric assay of Monobind Inc. (IEMA/ELISA) (100 North pointe Drive, Lake Forest, CA 92630 U.S.A.).

### 2.7. Statistical Analysis

Data were coded and entered in Excel 2013, then analyzed using XLSTAT 2014. Semen parameters and hormone levels were expressed as the median (5^th^–95^th^ percentiles). The Mann–Whitney U test was used to compare the reference and case groups.

Relationships between hormone levels and semen parameters were analyzed using Spearman's correlation test.

The evaluation of the parameters is based on the World Health Organization criteria.

Different aspects of semen analysis are discussed, such as enumeration of spermatozoa, viability, Kruger classification and interpretation of results.

Normozoospermia (reference group) corresponded to a total sperm count ≥20 million/ejaculate, progressive motility ≥32% (as defined in the 2010 WHO manual), and normal forms ≥15% (as defined in our laboratory).

A *p* value <0.05 was used to characterize significant results.

## 3. Results

### 3.1. Semen Characteristics and Hormone Level Distributions of the Study Population

We recruited a total of 240 men but only 156 of them actually provided blood and semen samples for analysis. Of these, 48 presented semen parameters within the reference limits and thus constituted the reference group (Normospermic). In contrast, 108 men presented at least one abnormal semen parameter (as defined above) and thus constituted the case group.

The mean age of our population was 37.101 ± 7.371 years and the majority classes were those aged 30–35 followed by those aged 40–45.

Our patients had on average one child and the type of infertility found was more secondary (53.846%).

Table 1 presents the semen characteristics and hormone level distributions of our population. We found a significant difference in the two groups regarding sperm concentration (*p* < 0.0001), total sperm count (*p* < 0.0001), progressive motility (*p* < 0.0001), vitality (*p* < 0.0001) and the percentage of normal forms (*p* = 0.043). No statistical difference has been found regarding age, number of children, days of abstinence, semen volume, pH, and viscosity (*p* > 0.05).

**Figure 1:**
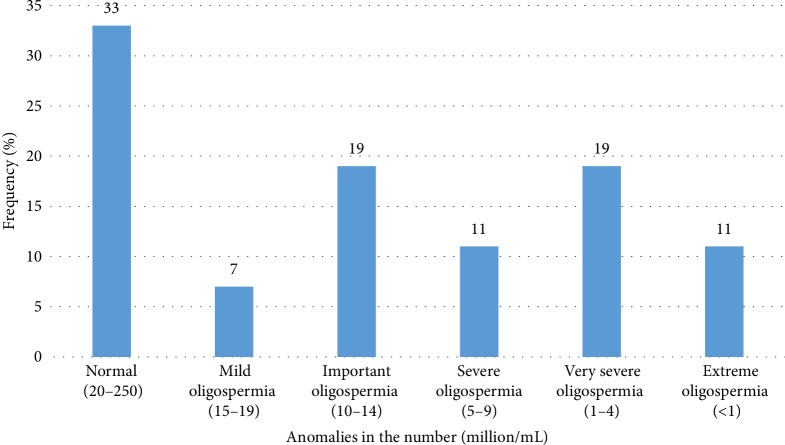
Distribution of the population according to anomalies in numbers.

The Figure 1 presents the distribution of the population based on the anomalies in the number. It appears that in terms of frequency, patients with significant oligospermia were execo with those with very severe oligospermia (19%).

**Figure 2:**
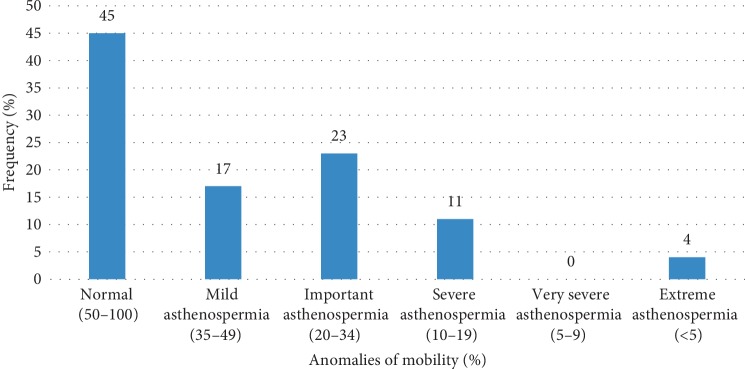
Distribution of the population according to mobility anomalies.

Concerning mobility abnormalities, Figure 2 shows that 23% of patients had significant asthenospermia, 17% a mild asthenospermia and 11% a severe asthenospermia.

**Figure 3:**
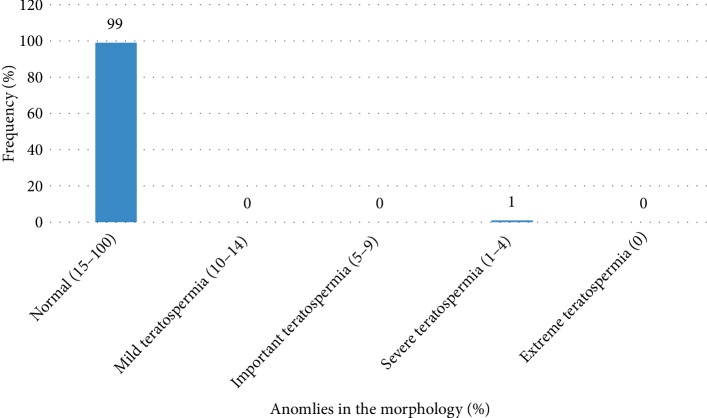
Distribution of the population based on morphology abnormalities.

The abnormalities of morphologies are presented in the Figure 3. We note that 99% of the patients had a normal sperm morphology.

**Figure 4:**
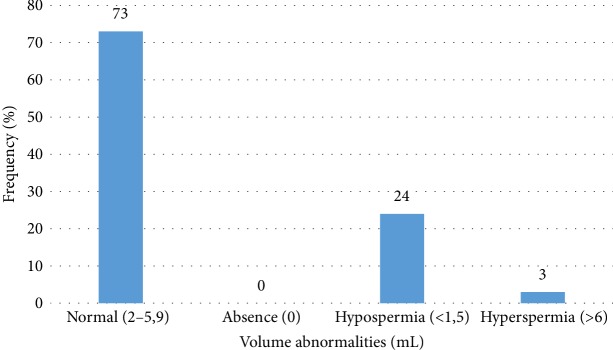
Distribution of the population according to volume anomalies.

The study of abnormalities in sperm volume (Figure 4) shows that 73% had a normal sperm volume, 24% were hypospermia, and 3% were hyperspermia.

Also, in a general point of view, about 46% of our infertile patients presented associations of the abnormalities cited above (morphology, mobility, number and volume abnormalities).

Table 2 shows the hormonal distributions of our population. We found that FSH was significantly higher in the case group (*p* = 0.020). In contrast, the level of Inhibin B was lower in the case group (*p* = 0.013).

The analysis of the hormone concentrations according to the age classes have shown a great increase of FSH with years (*p* < 0.0001) and a decrease of Inhibin B concentration (*p* = 0.328).

### 3.2. Correlations between Hormone Levels and Semen Parameters

Relationship between hormone levels and semen parameters have been studied in Table 3 and we have noticed in the **overall population**, a positive correlation between Inhibin B and sperm concentration (*p* = 0.028; *r* = 0.183), total sperm count (*p* < 0.0001; *r* = 0.307) and leucocytes (*p* = 0.006; *r* = 0.227); while a negative correlation between FSH and sperm concentration (*p* = 0.009; *r* = −0.217), total sperm count (*p* = 0.032; *r* = −0.179) and vitality (*p* = 0.018; *r* = −0.197). In the **reference group**, we noted a positive correlation between Inhibin B and the volume (*p* < 0.0001; *r* = 0.528) and a negative correlation between Inhibin B and the round cells (*p* = 0.038; *r* = −0.301). Finally, the **case group** showed positive correlation between Inhibin B and total sperm count (*p* = 0.006; *r* = 0.261) and leucocytes and a negative correlation between FSH and the total sperm count (*p* = 0.041; *r* = −0.197).

We found a strong and negative correlation between FSH and Inhibin B (*r* = −0.477; *p* < 0.0001) in the overall population, the normozoospermic group (reference group) (*r* = −0.367; *p* = 0.011), and the case group (*r* = −0.511; *p* < 0.0001).

We analyzed the relationship between semen parameters in our population.  Table 4 shows that, in the overall population, **positive** correlation has been found between the volume and Total sperm count (*r* = 0.235) and leucocytes (*r* = 0.187); the sperm concentration and total sperm count (*r* = 0.636), vitality (*r* = 0.540), mobility (*r* = 0.551), red blood cells (*r* = 0.232), and round cells (*r* = 0.232); the total sperm count and vitality (*r* = 0.525), motility (*r* = 0.520), normal forms (*r* = 0.249), and leucocytes (*r* = 0.238); the vitality and mobility (*r* = 0.969) and normal forms (*r* = 0.267); the motility and normal forms (*r* = 0.269); the normal forms and leucocytes (*r* = 0.231); the red blood cells and round cells (*r* = 0.614). On the other hand, we observed a **negative** correlation between the volume and pH (*r* = −0.215); the sperm concentration and leucocytes (*r* = −0.199); the total sperm count and pH (*r* = −0.185) and red blood cells (*r* = −0.391); the normal forms and red blood cells (*r* = −0.194); the red blood cells and leucocytes (*r* = −0.525); the leucocytes and round cells (*r* = −0.226) and viscosity (*r* = −0.192).

As shown in Table 5, in the reference population the correlation between semen parameters showed a **positive** correlation between volume and total sperm count (*r* = 0.286); the total sperm count and sperm concentration (*r* = 0.793); the vitality and motility (*r* = 0.799); leucocytes and round cells (*r* = 0.654). We also noticed a **negative** correlation between pH and sperm concentration (*r* = −0.349) and total sperm count (*r* = −0.296).

Concerning the correlation between semen parameters in the case group (Table 6), it appears a **positive** correlation between volume and the total sperm count (*r* = 0.193) and the leucocytes (*r* = 0.191); the pH and normal forms (*r* = 0.194); the sperm concentration and total sperm count (*r* = 0.597), vitality (*r* = 0.367) and motility (*r* = 0.420); the total sperm count and vitality (*r* = 0.347) and motility (*r* = 0.407); the red blood cells and round cells (*r* = 0.626); vitality and motility (*r* = 0.904). We also found a **negative** correlation between the pH and volume (*r* = −0.235) and leucocytes (*r* = −0.206); Leucocytes and viscosity (*r* = −0.214); total sperm count and red blood cells (*r* = −0.313); sperm concentration and leucocytes (*r* = −0.258); Leucocytes and red blood cells (*r* = −0.392).

## 4. Discussion

Previous studies have reported that circulating levels of specific reproductive hormones in men are associated with sperm quality parameters [4]. In particular, Inhibin B and FSH are considered markers of Sertoli spermatogenesis and cell function. It has even been suggested that measurement of both hormones in serum may be a substitute for measuring sperm quality or fecundability in epidemiological studies [4].

The current study analyzes blood levels of Inhibin B and FSH and their relationship with semen parameters.

In line with previous studies [11, 12, 22–29] we found that the infertile men's group was older than the reference group (with majority age classes from 30 to 35 followed by 40 to 45). This is probably the age at which men feel the need to start a family. More than 58% of our population had an advanced age of paternity (>35 years). In addition, we observed a great increase of FSH with years and a decrease of Inhibin concentration. The literature describes a positive and significant correlation between age and number of children, duration of infertility and marital status. This could be explained by the fact that as men age, testicular function and metabolism deteriorate when the testes undergo age-related morphological changes such as decreased germ cell counts, Leydig cells, and Sertoli, as well as structural changes, including shrinkage of seminiferous tubules [30, 31].

In men inhibin is secreted from the testis as a product of Sertoli cells involved in the regulation of FSH secretion [33]. We found a strong and negative correlation between FSH and Inhibin B in the overall population, the case and the reference group. This corroborates very well the findings of Barbotin et al. [5], Jensen et al. [32], and Von Eckardstein et al. [33], stating that FSH and Inhibin B are complementary tools in gonadal male conditions and andrological diagnostics [5]. In particular, FSH and Inhibin B together are more sensitive than either alone in predicting the histological status of the testis and the presence of sperm in bioptic tissue [5]. FSH which is a gonadotropin that is produced and secreted by the anterior pituitary, acts on Sertoli cells in the seminiferous tubules to initiate spermatogenesis. Sertoli cells secrete Inhibin B, which is a protein hormone. Thus, as found in our study, an association between Inhibin B and sperm concentration was to be expected, since the regulation of both factors is dependent upon Sertoli cell function [4, 5, 14].

In the overall population and the case group, Inhibin B was significantly and positively correlated with sperm concentration while FSH was negatively correlated to sperm concentration. We could not observe this correlation in the normospermic group. These findings confirmed those of Jorgensen et al.in 2010 [15], Kumanov et al.in 2006 [16], Myers et al.in 2008 [17], Sikaris et al. in 2005 [18] and Grunewald et al.in 2013 [19]. Moreover, Barbotin et al. explained that associations between hormone levels and sperm count are stronger in studies of the general population [32, 34] or infertile men [4, 16, 17, 35] than in studies of fertile men [15, 17]. They were precise that it could be due to the marked interindividual differences in sperm count seen in populations of primarily normozoospermic and/or fertile men. In total, categories of men (fertile, infertile, and normozoospermic) represented overlap in semen qualities, suggesting that Inhibin B and FSH were not mainly markers of fertility but rather markers of semen quality and thereby fecundity. In addition, It has been suggested that Inhibin B may be a better predictor of spermatogenesis than FSH [33]. The clinical value of the Inhibin B assay is emphasized by the fact that in contrast to FSH, Inhibin B is produced inside the testis and might reflect close interactions between Sertoli and germ cells [33]. Thus, Inhibin B levels correlate directly with spermatogenesis and reflect testicular sperm production.

Although the known association between sperm motility and sperm concentration [17], we could not observe in our population a correlation between sperm motility and Inhibin B and FSH levels but we did observe the correlation between sperm concentration and vitality and mobility both in the overall population and the case group but not in the normospermic group. Considering their overall population, Tomlinson et al. [20] and Karabulut et al. [21] also found a strong correlation between the sperm concentration, the motility and the morphology. Our results are different from Barbotin et al. who observed in their population a relationship between sperm motility and Inhibin B and FSH levels. But just like us they found a strong relationship between sperm concentration and vitality and mobility both in the overall population and the case group but not in the normospermic group. This could be explained by the fact that they included a very large number of individuals compared to our sample size.

## 5. Conclusion

At the end of this study, our results showed lower values of semen parameters in the case group especially concerning the sperm concentration, the progressive motility, the vitality, and the percentage of normal forms.

The correlation of Inhibin B and FSH to the sperm parameters highlights their role as markers of spermatogenesis. The combination of FSH and Inhibin B is currently the best predictor of the presence of spermatozoa.

## Figures and Tables

**Table 1:** Semen characteristics of the study population. Data are reported as the median (5^th^–95^th^ percentiles). Comparison between the reference group and the case group was performed using a Mann–Whitney *U* test.

Characteristics of the population	Overall population	Reference group	Case group	Normal values	*p*
Age (years)	37 (26–50)	37.6 (27–49.6)	36.5 (23.8–48.8)	NA	0.385
Number of children	1 (0–4)	1 (0–4)	1 (0–5)	NA	0.471
Abstinence (days)	3 (2–6)	3 (2–6)	3 (3–7)	3–5 days	0.441
Semen volume (mL)	2.92 (0.96–5.5)	2.95 (0.72–5.6)	2.8 (1.2–4.46)	≥2 mL	0.923
pH	8.31 (7.5–9)	8.32 (7.6–9)	8.3 (7.5–9)	≥7,2	0.594
Viscosity	1 (0–3)	1 (0–3)	1 (0–3)	0	0.431
Sperm concentration (million/mL)	14.8 (0.2–70.2)	10.8 (0.03–54.6)	39.6 (2.1–78.8)	≥20 × 10^6^/l	**<0.0001**
Total sperm count (million/ejaculate)	33 (0–194.08)	12.42 (0–109.1)	122.12 (14.83–300.28)	≥40 × 10^6^	**<0.0001**
Progressive motility (%)	43.2 (2–76)	35.3 (0–72.6)	64 (45.6–80.6)	≥50%	**<0.0001**
Vitality (%)	53 (10.8–85.2)	45.8 (9.4–81.4)	72 (56.4–86.6)	≥50%	**<0.0001**
Normal forms (%)	85 (67–94)	84 (65.8–94)	87 (74.8–92.6)	≥85%	**0.043**

*p* < 0.05 was considered to be statistically significant. The significant values are *p* < 0.0001 and *p* = 0.043.

**Table 2:** Distribution of the population according to the level of hormone FSH and Inhibin B.

Characteristics of the population	Overall population	Reference group	Case group	Normal values	*p*
Inhibin B (pg/mL)	139.1 (10.33–373.72)	153.2 (44.5–389.7)	125.94 (9.8–311.44)	>30 pg/mL	**0.013**
FSH (UI/L)	5.53 (0.2–29.4)	3.78 (0.1–34.93)	6.63 (0.24–28.62)	Young man: <5 UI/L	**0.020**
				Adult: 3–15 UI/L	
				Andropause (>40 years): 37–100 UI/L	

*p* < 0.05 was considered to be statistically significant. The significant values are *P* = 0.0013 and*P* = 0.020 which is less than 0.05.

**Table 3:** Relationship between hormone levels and semen parameters. Correlation was done using spearman correlation test.

Semen parameters	Population	Inhibin B (r; *p*)	FSH (r; *p*)
Semen volume (mL)	Overall population	0.139 (0.096)	−0.004(0.966)
	Reference group	**0.528^∗^ ( ** **p** < 0.0001** )**	−0.284(0.051)
	Case group	0.058 (0.553)	0.065 (0.504)
Viscosity	Overall population	−0.049 (0.562)	0.024 (0.779)
	Reference group	−0.045 (0.761)	0.102 (0.488)
	Case group	−0.083 (0.393)	−0.006 (0.949)
pH	Overall population	−0.117 (0.163)	0.159 (0.057)
	Reference group	−0.283 (0.052)	0.223 (0.127)
	Case group	−0.110 (0.258)	0.152 (0.116)
Sperm concentration (million/mL)	Overall population	**0.183^∗^ (0.028)**	−**0.217^∗^ (0.009)**
	Reference group	0.043 (0.771)	−0.001 (0.992)
	Case group	0.006 (0.948)	−0.113 (0.243)
Total sperm count (million/ejaculate)	Overall population	**0.307^∗^ ( ** **p** < 0.0001** )**	−**0.179^∗^ (0.032)**
	Reference group	0.245 (0.093)	−0.045 (0.762)
	Case group	**0.261^∗^ (0.006)**	−**0.197^∗^ (0.041)**
Vitality (%)	Overall population	0.103 (0.219)	−**0.197^∗^ (0.018)**
	Reference group	−0.159 (0.280)	0.024 (0.869)
	Case group	−0.056 (0.565)	−0.112 (0.249)
Progressive motility (%)	Overall population	0.072 (0.388)	−0.152 (0.069)
	Reference group	0.046 (0.755)	−0.062 (0.674)
	Case group	−0.032 (0.744)	−0.036 (0.709)
Normal forms (%)	Overall population	0.088 (0.296)	0.005 (0.956)
	Reference group	0.041 (0.780)	0.064 (0.664)
	Case group	−0.084 (0.387)	−0.010 (0.922)
Leucocytes	Overall population	**0.227^∗^ (0.006)**	0.025 (0.764)
	Reference group	−0.103 (0.486)	−0.036 (0.805)
	Case group	**0.207^∗^ (0.032)**	0.006 (0.949)
Red blood cells	Overall population	−0.073 (0.386)	−0.004 (0.958)
	Reference group	/	/
	Case group	−0.030 (0.757)	0.075 (0.437)
Round cells	Overall population	−0.027 (0.747)	−0.040 (0.635)
	Reference group	−**0.301^∗^ (0.038)**	0.024 (0.872)
	Case group	0.070 (0.473)	0.067 (0.492)

*p* < 0.05 was considered to be statistically significant. ^∗^Significant. The significant values in this table are*p* < 0.0001, *p* = 0.028, *p* = 0.006, *p* = 0.032, *p* = 0.038, *p* = 0.09, *p* = 0.018 and *p* = 0.041.

**Table 4:** Relationship between semen parameters in the overall population. Correlation was done using spearman correlation test.

Variables	Volume (mL)	Viscosity	pH	Sperm concentration (spz/ml)	Total sperm count (spz/ejaculate)	Vitality (1 h)%	Motility (1 h)%	Normal forms	Leucocytes	Red blood cells	Round cells
Volume (mL)	**1**	−0.087	−**0.215^∗^**	−0.122	**0.235^∗^**	0.138	0.160	0.097	**0.187^∗^**	−0.065	−0.005
Viscosity	−0.087	**1**	0.063	−0.025	−0.033	−0.027	−0.037	−0.122	−**0.192^∗^**	0.005	−0.087
pH	−**0.215^∗^**	0.063	**1**	−0.136	−**0.185^∗^**	−0.058	−0.069	0.121	−0.110	−0.043	−0.091
Sperm concentration (spz/ml)	−0.122	−0.025	−0.136	**1**	**0.636^∗^**	**0.540^∗^**	**0.551_^∗^_**	0.076	−**0.199^∗^**	**0.232^∗^**	**0.232^∗^**
Total sperm count (spz/ejaculate)	**0.235^∗^**	−0.033	−**0.185^∗^**	**0.636^∗^**	**1**	**0.525^∗^**	**0.520^∗^**	**0.249^∗^**	**0.238^∗^**	−**0.391^∗^**	−0.161
Vitality (1 h)%	0.138	−0.027	−0.058	**0.540^∗^**	**0.525^∗^**	**1**	**0.969^∗^**	**0.267^∗^**	0.044	−0.026	0.116
Motility (1 h)%	0.160	−0.037	−0.069	**0.551^∗^**	**0.520^∗^**	**0.969^∗^**	**1**	**0.269^∗^**	0.020	−0.018	0.148
Normal forms	0.097	−0.122	0.121	0.076	**0.249^∗^**	**0.267^∗^**	**0.269^∗^**	**1**	**0.231^∗^**	−**0.194^∗^**	−0.114
Leucocytes	**0.187^∗^**	−**0.192^∗^**	−0.110	−**0.199^∗^**	**0.238^∗^**	0.044	0.020	**0.231^∗^**	**1**	−**0.525^∗^**	−**0.226^∗^**
Red blood cells	−0.065	0.005	−0.043	**0.232^∗^**	−**0.391^∗^**	−0.026	−0.018	−**0.194^∗^**	−**0.525^∗^**	**1**	**0.614^∗^**
Round cells	−0.005	−0.087	−0.091	**0.232^∗^**	−0.161	0.116	0.148	−0.114	−**0.226^∗^**	**0.614^∗^**	**1**

*p* < 0.05 was considered to be statistically significant. ^∗^Significant. All the values marked with asterix (^∗^) are significant (*p* < 0.05).

**Table 5:** Relationship between semen parameters in the reference group. Correlation was done using spearman correlation test.

Variables	Volume (mL)	Viscosity	pH	Sperm concentration (spz/ml)	Total sperm count (spz/ejaculate)	Vitality (1 h)%	Motility (1 h)%	Normal forms	Leucocytes	Round cells
Volume (mL)	**1**	−0.078	−0.051	0.003	**0.286^∗^**	0.006	0.142	0.037	0.071	−0.151
Viscosity	−0.078	**1**	0.007	−0.118	−0.095	0.082	0.087	−0.145	−0.184	0.001
pH	−0.051	0.007	**1**	−**0.349^∗^**	−**0.296^∗^**	0.115	0.002	0.200	0.038	0.124
Sperm concentration (spz/ml)	0.003	−0.118	−**0.349^∗^**	**1**	**0.793^∗^**	−0.051	0.110	−0.129	0.115	−0.010
Total sperm count (spz/ejaculate)	**0.286^∗^**	−0.095	−**0.296^∗^**	**0.793^∗^**	**1**	−0.245	−0.031	−0.087	0.208	0.007
Vitality (1 h)%	0.006	0.082	0.115	−0.051	−0.245	**1**	**0.799^∗^**	−0.211	−0.027	0.211
Motility (1 h)%	0.142	0.087	0.002	0.110	−0.031	**0.799^∗^**	**1**	−0.072	0.104	0.267
Normal forms	0.037	−0.145	0.200	−0.129	−0.087	−0.211	−0.072	**1**	−0.045	−0.113
Leucocytes	0.071	−0.184	0.038	0.115	0.208	−0.027	0.104	−0.045	**1**	**0.654^∗^**
Round cells	−0.151	0.001	0.124	−0.010	0.007	0.211	0.267	−0.113	**0.654^∗^**	**1**

*p* < 0.05 was considered to be statistically significant. ^∗^Significant. All the values marked with asterix (^∗^) are significant (*P* < 0.05).

**Table 6:** Relationship between semen parameters in the case group. Correlation was done using spearman correlation test.

Variables	Volume (mL)	Viscosity	pH	Sperm concentration (spz/ml)	Total sperm count (spz/ejaculate)	Vitality (1 h)%	Motility (1 h)%	Normal forms	Leucocytes	Red blood cells	Round cells
Volume (mL)	**1**	−0.072	−**0.235^∗^**	−0.109	**0.193^∗^**	0.101	0.121	0.084	**0.191^∗^**	−0.054	0.043
Viscosity	−0.072	**1**	0.061	0.059	−0.005	0.084	0.043	−0.131	−**0.214^∗^**	0.013	−0.112
pH	−**0.235^∗^**	0.061	**1**	−0.021	−0.179	−0.083	−0.072	**0.194^∗^**	−**0.206^∗^**	−0.051	−0.078
Sperm concentration (spz/ml)	−0.109	0.059	−0.021	**1**	**0.597^∗^**	**0.367^∗^**	**0.420^∗^**	0.070	−**0.258^∗^**	0.146	0.153
Total sperm count (spz/ejaculate)	**0.193^∗^**	−0.005	−0.179	**0.597^∗^**	**1**	**0.347^∗^**	**0.407^∗^**	0.141	0.145	−**0.313^∗^**	−0.125
Vitality (1 h)%	0.101	0.084	−0.083	**0.367^∗^**	**0.347^∗^**	**1**	**0.904^∗^**	0.089	−0.079	0.059	0.112
Motility (1 h)%	0.121	0.043	−0.072	**0.420^∗^**	**0.407^∗^**	**0.904^∗^**	**1**	0.163	−0.111	0.045	0.142
Normal forms	0.084	−0.131	**0.194^∗^**	0.070	0.141	0.089	0.163	**1**	0.146	−0.111	−0.109
Leucocytes	**0.191^∗^**	−**0.214^∗^**	−**0.206^∗^**	−**0.258^∗^**	0.145	−0.079	−0.111	0.146	**1**	−**0.392^∗^**	−0.158
Red blood cells	−0.054	0.013	−0.051	0.146	−**0.313^∗^**	0.059	0.045	−0.111	−**0.392^∗^**	**1**	**0.626^∗^**
Round cells	0.043	−0.112	−0.078	0.153	−0.125	0.112	0.142	−0.109	−0.158	**0.626^∗^**	**1**

*p* < 0.05 was considered to be statistically significant. ^^∗^^Significant. All the values marked with asterix (^∗^) are significant (*P* < 0.05).

## Data Availability

The biological data used to support the findings of this study are available from the corresponding author upon request.
